# The role of silicon in physiology of the medicinal plant (*Lonicera japonica L.*) under salt stress

**DOI:** 10.1038/srep12696

**Published:** 2015-08-03

**Authors:** Zhao Gengmao, Li Shihui, Sun Xing, Wang Yizhou, Chang Zipan

**Affiliations:** 1 College of Resources and Environmental Sciences, Nanjing Agricultural University, Nanjing, Jiangsu Province, P.R. China; 2 Jiangsu Provincial Key Lab of Marine Biology, Nanjing Agricultural University, Nanjing, Jiangsu Province, P.R. China

## Abstract

Silicon(Si) is the only element which can enhance the resistance to multiple stresses. However, the role of silicon in medicinal plants under salt stress is not yet understood. This experiment was conducted to study the effects of silicon addition on the growth, osmotic adjustments, photosynthetic characteristics, chloroplast ultrastructure and Chlorogenic acid (CGA) production of Honeysuckle plant (*Lonicera japonica L.*) under salt-stressed conditions. Salinity exerted an adverse effect on the plant fresh weight and dry weight, whilst 0.5 g L^−1^ K_2_SiO_3_·nH_2_O addition obviously improved the plant growth. Although Na^+^ concentration in plant organs was drastically increased with increasing salinity, higher levels of K^+^/Na^+^ ratio was obtained after K_2_SiO_3_·nH_2_O addition. Salinity stress induced the destruction of the chloroplast envelope; however, K_2_SiO_3_·nH_2_O addition counteracted the adverse effect by salinity on the structure of the photosynthetic apparatus. K_2_SiO_3_·nH_2_O addition also enhanced the activities of superoxide dismutase and catalase. To sum up, exogenous Si plays a key role in enhancing its resistance to salt stresses in physiological base, thereby improving the growth and CGA production of Honeysuckle plant.

Most of the environmental constraints drastically decrease plant growth^1^. Among abiotic threats, salinity is of special concern. Salinization is a global problem, and has attracted more attention of the international academic community. It is most common in arid and semi-arid areas all over the world and responsible for a substantial decline in crop yield^2^. Therefore, the efforts to screen economically salt-tolerant crops or plants bear remarkable importance for sustainable agriculture. Honeysuckle (*Lonicera japonica L*.), as an economic plant, is widely appreciated as tea beverage in Asia and Europe because of its unique aroma and flavour^3^. Moreover, it is commonly used in traditional Chinese medicine (TCM) for the treatment of arthritis, diabetes mellitus, enteritis and fever^4^,^5^. Pharmacological studies have shown that the constituents of honeysuckle have a broad spectrum of biological activity, such as antiviral, antipyretic and hepatoprotective effects^6^,^7^. For this reason, honeysuckle is being cultivated in salt-affected areas, such as tidal flats in coastal zones, for its specific therapeutic benefits.

Generally, plants adopt a number of strategies to cope with adverse effects of salt stress, in which osmotic adjustment is an important one^8^. Plants undergo osmotic adjustments under abiotic stress condition by avoiding Na^+^ and Cl^−^ uptake which causes ion toxicity and accumulating K^+^ , Ca^2+^ , enabling plants to withstand salinization^9^. The amount of Na^+^ absorbed exceeds that of Na^+^ extruded through the plasma membrane (PM) cation channels, leading to excessive Na^+^ -toxicity events, such as damage to these systems that absorb and utilize Ca^2+^ and K^+^ ions. Damage to these systems negatively affects plant growth and development, since Ca^2+^ and K^+^ are essential for various physiological and biochemical processes^10^,^11^,^12^,^13^.

Additionally, a decrease in photosynthesis capacity is very common in salt stressed plant, mostly due to a low osmotic potential of the soil solution (osmotic stress), specific ion effects (salt stress), nutritional imbalances or more usually, a combination of these factors^14^. Salinity influences photosynthetic capacity and its effects vary with the salt concentration, duration of stress and the cultivar used. Photosynthetic ability of some plant species reduced in the presence of salinity^15^,^16^, while that of other species reduced only at high stress and long term stress^17^.

Also, salinity is one of the major stresses responsible for changes in metabolic activity of plants. Plants have evolved several adaptive mechanisms to handle with the salinity in their environments, but the understanding of these mechanisms still remains incomplete. It has been shown that reactions to salt and drought stress might be responsible for the increase or decrease in the content of relevant natural products; however, scientific background in this field is still rare^18^. Medicinal plants under salt stress conditions accumulate higher concentrations of secondary compounds than control plants which are cultivated under standard conditions. Chlorogenic acid (CGA) is a type of polyphenol and one kind of secondary metabolites, which has anti-inflammatory, anti-oxidative and anticancer properties^19^,^20^,^21^. CGA could inhibit inflammatory cell infiltration, notably neutrophil recruitment into lung^22^ and inhibit inflammatory cytokines release through suppressing nuclear factor kappa B (NF-kB) activation^23^. Keeping this in view, accumulation of CGA in the Honeysuckle plant under salt stress conditions was studied in this experiment.

Several investigators have reported that silicon enhanced the salt tolerance of wheat^24^, barley^25^,^26^, maize^27^ and tomato (Solanum lycopersicum L. cv. Hong mei)^28^. However, its role in plant biology has been poorly understood and the attempts to associate Si with metabolic or physiological activities have been inconclusive^29^.

The objective of this study was to elucidate the salt-tolerant mechanisms of the Honeysukle plant on physiological bases, therefore revealing the role of Silicon in the amelioration of salt hazards and enhancement of secondary metabolite in the medicinal plant.

## Materials and methods

### Experimental layout

The experiment was conducted in a greenhouse at College of Resources and Environmental Sciences, Nanjing Agricultural University, during September 14 to October 25, 2013. Honeysuckle (*Lonicera japonica L.*) seedlings were obtained from BoZhou city, AnHui Province. Nearly 30-day-old nursery seedlings were transplanted into plastic pots (24 cm in diameter, 18 cm in depth), one seedling per pot. The pots were filled with clean sand and each pot was watered with 1.5 L Hoagland Solution. The experiment was arranged in a completely randomized design (CRD) and each treatment had three replications.

Plants were subjected to salt stress by adding sodium chloride (NaCl) solutions, with the calculated amount of NaCl and K_2_SiO_3_·nH_2_O dissolved in Hoagland Solution. The experiment consisted of three treatments involving varying salinity levels and three levels of K_2_SiO_3_·nH_2_O in sand (Table 1).To minimize salt shock, NaCl concentration was raised stepwise in aliquots of 50 mM every other day until the final salinity levels were achieved. The greenhouse temperature and relative humidity varied 25–30 °C and 50–80%, respectively for the entire growth period. Photosynthesis is determined at the 30^th^ day after salt treatment, respectively. Plants were sampled (the whole plant) at the 40^th^ day after salt treatment to assess the growth, osmotic adjustments, chloroplast ultrastructrue and chlorogenic acid (CGA) accumulation. After washing and drying, leaves were frozen in liquid N_2_ and stored at −80 °C until biochemical analysis.

### Growth status analysis

FW is the fresh weight, and DW is the dry weight after oven-drying samples at 70 °C until constant weight.

### Elemental analysis

Leaves, stems and roots were separated and washed with ultra-pure water to eliminate salts in the surface of these samples. Subsequently they were dried at 70 °C until constant weight and grinded in a stoneware mortar, from which were taken 100 mg for acid digestion. This procedure was carried out in Teflon reactors using an acid mixture of HNO_3_:HClO_4_ (3:1, v/v) during 2 h at 280  °C until the liquid was almost transparent. After cooling overnight, the digestion products were diluted with distilled water to a total volume of 100 ml. Elemental concentrations in the extracts were determined by ICP-AES (Inductively Coupled Plasma Atomic Emission Spectrometry, Optima 2100DV, Perkin Elmer, USA). Si was determined following the method of Nayar *et al.*^30^.

### Transmission electron microscope assay

The fifth fully expanded leaves, numbered basipetally, were sampled for ultramicroscopic observation on day 30 after the onset of the final concentration of salt treatment. For transmission electron microscope, fresh leaves were cut into 2 segments of 1 mm each and placed immediately in a freshly prepared the stationary liquid of 2.5% (w/v) glutaraldehyde in 50 mM sodium phosphate buffer (PH 7.4). The segments were then de-gassed and fixed under vacuum for 4 h at room temperature. After washing in the same buffer, they were post-fixed in 1% (w/v) osmium tetroxide (in the same buffer) for 1 h. After fixation, the samples were rinsed three with the same buffer for 15 min. Rapid dehydration was accomplished by placing the sample in 50% ethanol for 30 min, followed by changes in 70%, 90%, 95%, 100% ethanol (30 min each). The specimens were then placed in low-viscosity epoxy resin. UL-trathin sections (80 nm) were mounted on uncoated nickel grids (300 meshes) and sequentially stained with uranyl acetate and lead citrate before being examined at 80 kV under a transmission electron microscope (H-7650, JEOL, Japan).

### The determination of leaf photosynthesis

The tested photosynthetic parameters included photosynthetic rate (A), Transpiration rate (E), stomatal conductance (gs) and Intercellular CO_2_ concentration (Ci). They were determined by portable photosynthesis system Li-6400XT (Li-cor Inc., Lincolin, NE, USA). The PAR was provided by a red/blue light-emitting diode (model Li 6400-02B; Li-cor Inc., NE, USA) and was set at 400 or 700 umol photons m-2s-1 to match the PAR used when exposing plants to control or high light conditions, respectively. The atmospheric CO_2_ concentration was controlled with a CO_2_ injection system controlled by the Li-6400 (Li-cor Inc., Lincolin, NE, USA). The cuvette temperature was maintained at 25 °C with a relative humidity of 65%.

### Pre-segmentation of CGA

Leaves washed with ultra-pure water to eliminate salts in the surface of these samples. Subsequently were dried at 70 °C until constant weight and grinded in a stoneware mortar, from which were taken 1.00 g for pre-segmentation. 1.0 g of leaf tissue of both control and stressed plants was homogenized in 14 mL 60% (v/v) ethylalcohol, and the pH of homogenate was 4.0. Homogenate was extracted in an ultrasonic cleaner (Xokeji, NJ, CHN) 15 min, then leached by Buchner funnel using a 0.45 um filter membrane.

Estimation of CGA in the Honeysuckle plant (*Lonicera japonica L*.) was done using HPLC. Chromatography was done in RP 18 columns with a flow rate of 1.0 mL min^−1^ using 1% phosphate buffer in HPLC acetonitrile (87:13) as carrier solvent. The above step was repeated for three times, and then collected and amalgamated the filter liquor for HPLC analysis. The sample load was 20 uL. Column temperature was maintained at 25 °C and absorbance was measured at 327 nm.

### Measurement of Antioxidant enzyme activities

The activity of SOD was determined following Giannopolitis and Ries^31^ by measuring its ability to inhibit the photoreduction of nitroblue tetrazolium (NBT). Activities of catalase (CAT) and peroxidase (POD) were appraised using the method of Chance and Maehly^32^ with some modification. The CAT reaction solution (3 ml) contained 50 mM phosphate buffer (PH 7.0), 5.9 mM H2O2, and 50 ul enzyme extract. Then the reaction was initiated by adding the enzyme extract. Changes in absorbance of the reaction solution at 240 nm were read every 40 s. One unit CAT activity was defined as an absorbance change of 0.01 units per min. The POD reaction solution (3 ml) contained 50 mM phosphate buffer (PH 6.0), 20 mM guaiacol, 40 mM H2O2, and 30 ul enzyme extract. Changes in absorbance of the reaction solution at 470 nm were determined after every 15 s. The activity of each enzyme was expressed on protein basis.

### Statistical analysis

Data were statistically analyzed using analysis of variance (ANOVA) by SPSS v.20 for Windows, and presented as treatment mean ± SE of three measurements. Tukey’s multiple range tests were calculated for the significant data at P < 0.05.

## Results

### Plant growth status

The effect of K_2_SiO_3_·nH_2_O addition on the fresh weight and dry weight of Honeysuckle plant (Lonicera japonica L.) was shown in Table 2. The highest value of the fresh weight and dry weight for no salt treatments was obtained in 0.5 g L^−1^ K_2_SiO_3_·nH_2_O addition treatments, implying that the optimum dose of K_2_SiO_3_·nH_2_O addition (0.5 g L^−1^ K_2_SiO_3_·nH_2_O) did enhance the plant growth. The same trends, however, were observed in 0.5 g L^−1^ K_2_SiO_3_·nH_2_O × 100 mM NaCl treatment and only for plant fresh weight. Additionally, higher concentration of K_2_SiO_3_·nH_2_O addition (1.0 g L^−1^) exerted no effect on the plant fresh weight and dry weight. As a whole, NaCl concentration of <200 mM did not affect the plant growth, suggesting the tested variety of Honeysuckle itself was a more salt-tolerant one. These results implied that K_2_SiO_3_·nH_2_O addition with 0.5 mg L^−1^ promoted the growth of the Honeysuckle, therefore increasing salt tolerance of the plant to a certain extent.

### Osmotic adjustments

Salt stress significantly affected the ionic uptake and distribution in the plant organs (Table 3). Na^+^ concentration in leaves, stems and roots was significantly increased under salt-stressed conditions as compared with the control. However, this increment in Na^+^ concentration was partially counteracted in 100 mM NaCl treatment by 0.5 mg L^−1^ K_2_SiO_3_·nH_2_O application. Additionally, an interesting phenomenon was investigated that Na^+^ concentration in plant organs was much lower than K^+^ concentration, indicating that selective absorption of K^+^ over Na^+^ (high S_K+/Na+_) could be one of the mechanism for this plant adapting to salt stress. Salt stress also caused the declined Ca^2+^ uptake by plant; whereas 1 g L^−1^ K_2_SiO_3_·nH_2_O application increased the Ca^2+^ concentration to a high level of approximately equal to that in the control treatment. Thus, it was concluded that K_2_SiO_3_·nH_2_O application played an important role in osmotic adjustments, subsequently enhancing the salt tolerance of the Honeysuckle plant.

### Chloroplast ultrastructure

**Figure 1:**
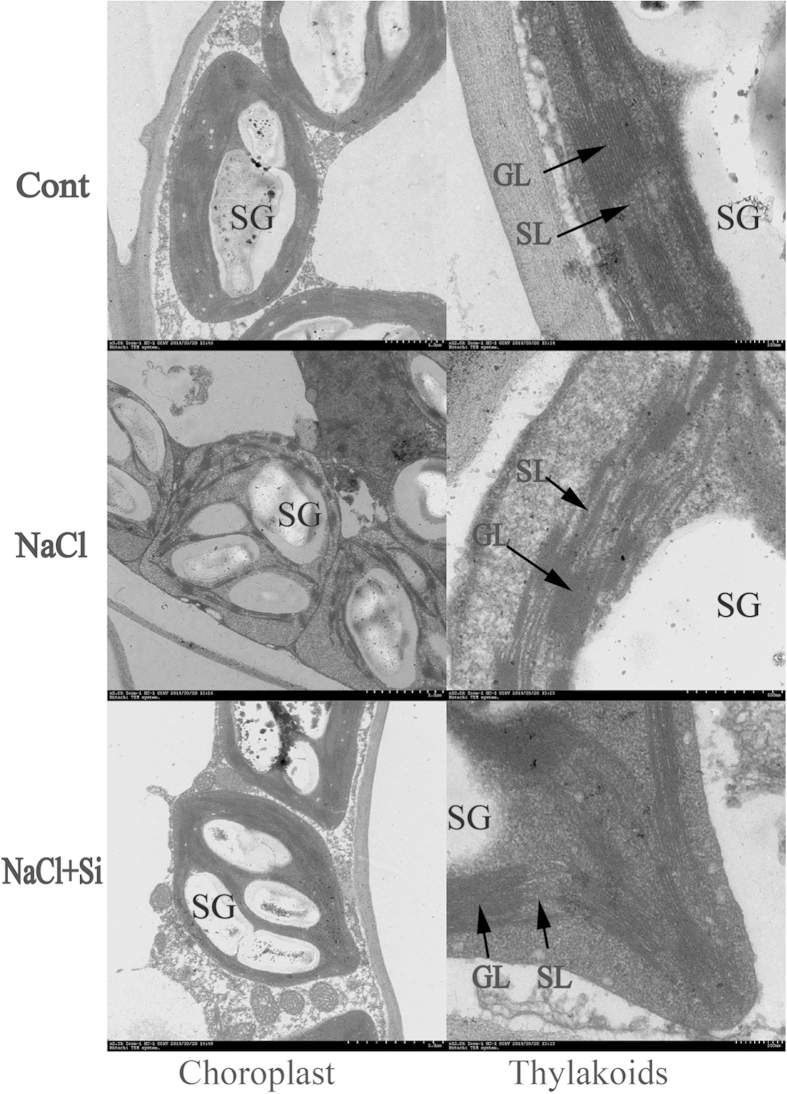
The effects of salt stress with and without exogenous remission of K_2_SiO_3_·nH_2_O on the chloroplast ultrastructure in leaves of Honeysuckle (*Lonicera japonica L.*). (**a**) Cont; 0 mM NaCl + 0 g L^−1^ K_2_SiO_3_·nH_2_O, (**b**) NaCl; 200 mM NaCl + 0 g L^−1^ K_2_SiO_3_·nH_2_O, (**c**) NaCl + Si; 200 mM NaCl + 0.5 g L^−1^ K_2_SiO_3_·nH_2_O. SL, stroma lamella; GL, grana lamellae; SG, starch grain.

There were no significant differences in ultrastructure of chloroplasts between the control and control plus remission of K_2_SiO_3_·nH_2_O (Fig. 1). Salt stress induced alterations in the structure of chloroplasts as compared to the control. The chloroplasts in salt-stressed plants were separated from the plasmamembrane, where chloroplasts were in closely contact with the membrane. The envelope membrane in the NaCl treated samples appeared to be ruptured. However, exogenous remission of K_2_SiO_3_·nH_2_O alleviated the structural changes of chloroplasts induced by salt stress. Remission of K_2_SiO_3_·nH_2_O maintained a well-preserved internal lamellar system in the chloroplasts of salt-stressed leaves and the chloroplasts contained less osmiophilic plastoglobuli. These results suggested that K_2_SiO_3_·nH_2_O application helped to maintain the integrity of chloroplast ultrastructrue, thus executed a normal physiological functions of the plant exposed to salt stress.

### Plant photosynthesis

The changes of photosynthetic characteristics in leaf Honeysuckle were shown in Table 4. Salt stress markedly reduced the photosynthetic rate in Honeysuckle leaves, whilst K_2_SiO_3_·nH_2_O application with the concentration of 1.0 g L^−1^ unexpectedly increased the photosynthetic rate. Similarly, the transpiration rate and stomatal conductance were significantly decreased with increasing NaCl concentrations no matter what K_2_SiO_3_·nH_2_O was applied or not. However, 0.5 g L^−1^ K_2_SiO_3_·nH_2_O addition appeared no effect on the plant photosynthesis, transpiration rate and stomatal conductance, but even inhibited them in a great deal.

### Secondary metabolite of CGA

**Figure 2:**
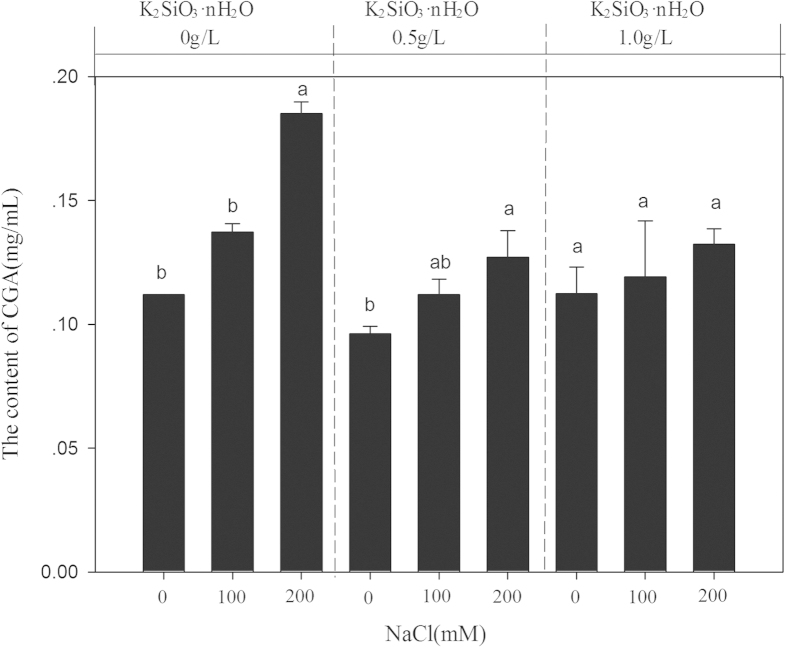
Secondary metabolite of CGA quantified in the leaves of Honeysuckle plant (*Lonicera japonica L.*) exposed to salt stress. Different letters within each column indicated significant difference among treatments at the p < 0.05 level. Three independent HPLC profiles were run for each sample and value represents mean ± S.E.

**Figure 3:**
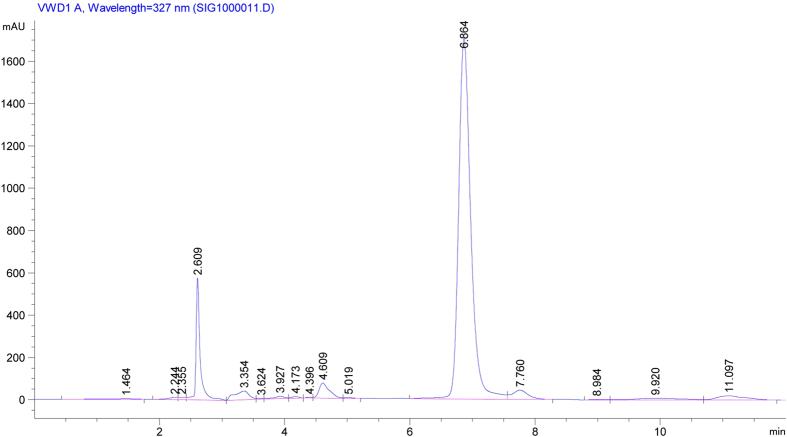
Representative chromatogram of prepared extract of Honeysuckle leaves (*Lonicera japonica L.*) grown in 200 mM NaCl concentrations with retention time of 7 min for CGA measurement.

CGA content in leaf Honeysuckle increased significantly with increasing salinities, as compared with the control (Fig. 2). With exogenous application of K_2_SiO_3_·nH_2_O, CGA content was gradually decreased, suggesting that CGA production in the plant leaves was hindered although K_2_SiO_3_·nH_2_O application alleviated plant injury induced by salt stress. Moreover, no significant difference was observed in CGA content of leaf Honeysuckle among all salt-stressed treatments when 1.0 g L^−1^ K_2_SiO_3_·nH_2_O was applied. In this experiment, the representative chromatogram with retention time for CGA was 7 min (Fig. 3).

## Discussion

Silicon has been extensively shown to increase crop yield and stress tolerance^33^. In this study, we found that K_2_SiO_3_·nH_2_O addition resulted in an improvement in the growth of salt-stressed Honeysuckle (*Lonicera japonica L*.) seedlings. These findings were comparable with observations in other crops, for example sorghum studied by Yin^34^, in which the application of silicon alone had no effects upon sorghum growth, while it partly reversed the salt-induced reduction in plant growth. In fact, silicon application resulted in the improvement of growth in many plants exposed to salt stresses that is often associated with many aspects, such as salt concentration, plant salt tolerance in addition to the plant species or genotypes. For rice plant, added Si can increase rigidity of the mature leaves, which have a rougher texture and are held more horizontally, delays leaf senescence and increases chlorophyll content and ribulose-bisphosphate carboxylase activity^35^. In this experiment, it was evident that Si uptake by the plant increased with increasing concentration of exogenous K_2_SiO_3_·nH_2_O, while inhibited by increasing salinity of hydroponic solution, suggesting Si may be involved in the metabolic or physiological activity in Honeysuckle exposed to salt stress (Table 5).

Maintaining structural integrity and orderliness of chloroplast is necessary in the conversion of light energy for photosynthesis. It was reported that many stressors led to the decrease in the photochemical efficiency and electron transport activity that might be associated with the changes of the structure of photosynthetic apparatus^36^. In the present study, we observed that exogenous Si decreased the separation of plasma membrane from plasmolysis. Moreover, it commendably maintains the integrity of the plasma membrane. These results demonstrated that silicon was involved in protecting the photosynthetic apparatus. Our results also showed that photosynthetic activity in Honeysuckle (*Lonicera japonica L.*) was affected by salinity, and the extent of the reduction was dependent on the salt strength. Zollinger^37^ found a similar reduction in photosynthetic rate and stomatal conductance in *E. purpurea* irrigated with increasing concentrations of salinity up to 5 gL^−1^. Reduction in photosynthetic rate under salinity can be attributed to both stomatal and non stomatal limitations^38^. In this study, we found a strong negative correlation between stomatal conductance and Na^+^ concentrations both in the aboveground parts and roots, which indicates that plants tend to close their stomatal conductance as a result of specific-ion accumulations. This reduction in stomatal conductance led subsequently to a reduction in photosynthetic rate as indicated by the high correlation between stomatal conductance and photosynthetic rate. Gong *et al.*^39^ reported that silicon did not enhance the K^+^ content of rice under salt stress. This is in agreement with our results that silicon had no effect on the K^+^ concentration. It is well known that high concentrations of Na^+^ can inhibit K^+^ and Ca^2+^ uptake through antagonism between these ions^40^. This fact can be attributed to their physicochemical similarity, which promotes ionic competition for binding sites on membrane transporters^41^. Further studies on antioxidant systems showed that the activities of superoxide dismutase, and catalase were continuously enhanced with increasing salinity, thereby protecting the Honeysuckle plants from oxidative injury (Table 6). Moreover, 0.5 g L^−1^ K_2_SiO_3_·nH_2_O addition did increase the activities of superoxide dismutase and catalase, demonstrating exogenous Si played an important role in these two enzymes.

The data recorded elevated CGA content under both non-silicon kinds of salinity when compared to the with-silicon saline conditions plants suggesting their probable role in the secondary metabolite of CGA. Many hypotheses suggest that the synthesis of secondary metabolites, including saponins, a large diverse group of secondary metabolites defined as amphipathic glycosides in which glycosyl residues are attached to tritrtpenoid (triterpeneor or teroidal) aglycon^42^, is a plant response to environmental factors and part of an adaptative strategy leading to tolerance to abiotic stresses. Just showed as our results, the content of secondary metabolite of CGA is highest at 200 Mm NaCl level without any remission of silicon, indicating that fluctuations in the content of CGA resulting from salinity changes in environmental circumstances might be expected. In fact, the level of CGA like other plant secondary metabolites, can be significantly influenced by the physiological status of the plant as well as the external factors, both abiotic and biotic, including temperature, availability of water and interactions with pathogens and parasites. However, CGA biosynthesis and accumulation by plant tissues in response to saline conditions in plant habitat are still not clear, so the study on Honeysuckle plant (*Lonicera japonica L.*) might have considerable merit in it.

In this paper, the salt-tolerant mechanisms of the Honeysukle plant on physiological and biochemical bases have been elucidated, which is well consistent with the study aims. The role of silicon in genomics and proteome of the medicinal plant (*Lonicera japonica* L.) under salt stress will be studied further in future.

## Conclusions

Plant salt tolerance is a multifaceted physiological trait. In line with physiological observations, medicinal Honeysuckle plant (*Lonicera japonica L*.) adapted to salt stress under K_2_SiO_3_·nH_2_O addition is mainly relied on ionic osmotic adjustment, improvement of superoxide dismutase and catalase activities and stability of chloroplasts ultrastructures. These results clearly highlighted the role of Si in protecting the plant against the hazardous effect of salinity. Also, this study provided the evidence that high salt stress affected the entire quantity of secondary metabolite of CGA produced. Thus, regulation of salinity through K_2_SiO_3_·nH_2_O addition could be a promising way to obtain better growth and considerable secondary metabolite of the medicinal plant.

## Additional Information

**How to cite this article**: Gengmao, Z. *et al.* The role of silicon in physiology of the medicinal plant (*Lonicera japonica L.*) under salt stress. *Sci. Rep.* **5**, 12696; doi: 10.1038/srep12696 (2015).

## Figures and Tables

**Table 1:** Nine treatments (NaCl × K_2_SiO_3_·nH_2_O) designed in this experiment.

**NO.**	**NaCl (mM)**	**K_2_SiO_3_·nH_2_O (g/L)**
1	0	0
2	0	0.5
3	0	1.0
4	100	0
5	100	0.5
6	100	1.0
7	200	0
8	200	0.5
9	200	1.0

**Table 2:** Effect of K_2_SiO_3_·nH_2_O addition on the fresh weight and dry weight of Honeysuckle plant (*Lonicera japonica L.*) under NaCl-stressed conditions.

The potency of K_2_SiO_3_·nH_2_O (g L^−1^)	**Plant fresh weigh (g plant**^−**1**^**) Salt levels (mM of NaCl)**				
	**Control (0 mM)**	**100**		**200**	
	**Mean ± S.E**	**Mean ± S.E**	**Percent of control**	**Mean ± S.E**	**Percent of control**
0	17.34 ± 2.99^aB^	15.61 ± 2.68^aB^	90.02	13.54 ± 1.95^aA^	78.09
0.5	25.41 ± 2.21^aA^	22.01 ± 3.30^aA^	86.70	16.45 ± 3.20^aA^	64.74
1.0	17.00 ± 1.75^aB^	13.47 ± 3.25^aB^	79.24	13.15 ± 3.61^aA^	77.35
The potency of K_2_SiO_3_·nH_2_O (g L^−1^)	Plant dry weigh (g plant^−1^) Salt levels (mM of NaCl)				
	Control (0mM)	100		200	
	Mean ± S.E	Mean ± S.E	Percent of control	Mean ± S.E	Percent of control
0	5.46 ± 0.54^aB^	4.53 ± 1.03^aA^	82.97	4.28 ± 0.51^aA^	78.31
0.5	7.12 ± 1.02^aA^	5.26 ± 1.23^aA^	73.90	5.04 ± 2.50^aA^	70.86
1.0	4.63 ± 0.72^aB^	4.40 ± 1.14^aA^	95.03	4.09 ± 1.76^aA^	88.46

The values represent the means of three replicates. The means followed by the same letters (lower-case letters within lines and upper-case letters within columns, respectively) did not differ significantly at 5% probability by Tukey’s test.

**Table 3:** Effect of K_2_SiO_3_·nH_2_O addition on the ion distribution within Honeysuckle (*Lonicera japonica L.*) plant under NaCl-stressed conditions.

**The potency of K_2_SiO_3_·nH_2_O (g L^−1^)**	**Na**^+^ **Concentration ((mg g**^−**1**^ **dwt) Salt levels (mM of NaCl)**					
	**Control (0mM)**		**100**		**200**	
	**Leaves and stems**	**Roots**	**Leaves and stems**	**Roots**	**Leaves and stems**	**Roots**
0	0.66 ± 0.05aA	0.45 ± 0.07bB	5.16 ± 0.12aA	6.65 ± 0.14aA	4.58 ± 0.28bA	8.95 ± 0.00aAB
0.5	0.66 ± 0.05cA	0.56 ± 0.06cB	3.16 ± 0.29bB	4.52 ± 0.18bB	5.60 ± 0.16aA	10.46 ± 0.08aA
1.0	0.11 ± 0.00cB	0.96 ± 0.08bA	5.39 ± 0.06aA	6.34 ± 0.21aA	4.52 ± 0.12bA	7.78 ± 0.52aB
The potency of K_2_SiO_3_·nH_2_O (g L^−1^)	K^+^ Concentration ((mg g^−1^ dwt) Salt levels (mM of NaCl)					
	Control (0 mM)		100		200	
	Leaves and stems	Roots	Leaves and stems	Roots	Leaves and stems	Roots
0	19.36 ± 1.16^bA^	39.64 ± 0.88^aA^	23.30 ± 0.57^aA^	37.71 ± 0.92^aA^	18.58 ± 1.19^bB^	33.65 ± 0.34^bA^
0.5	22.44 ± 0.13^aA^	37.42 ± 0.89^aA^	20.62 ± 2.79^aA^	40.08 ± 1.25^aA^	22.97 ± 0.92^aA^	40.54 ± 1.07^aA^
1.0	8.56 ± 0.004^cB^	39.39 ± 2.92^aA^	24.86 ± 0.64^aA^	40.30 ± 0.69^aA^	21.53 ± 1.42^bAB^	38.12 ± 2.06^aA^
The potency of K_2_SiO_3_·nH_2_O (g L^−1^)	S_K_^+^_/Na_^+^ Salt levels (mM of NaCl)					
	Control (0 mM)		100		200	
	Leaves and stems	Roots	Leaves and stems	Roots	Leaves and stems	Roots
0	31.64 ± 0.16^aB^	87.69 ± 9.02^aA^	4.60 ± 0.04^bB^	5.52 ± 0.27^bC^	4.06 ± 0.05^cA^	3.81 ± 0.09^bB^
0.5	34.01 ± 2.23^aAB^	71.45 ± 7.39^aA^	6.31 ± 0.22^bA^	8.88 ± 0.53^bA^	4.00 ± 0.11^bA^	3.90 ± 0.11^bB^
1.0	43.53 ± 4.20^aA^	76.47 ± 1.96^aA^	4.43 ± 0.19^bB^	6.66 ± 0.55^bB^	4.77 ± 0.24^bA^	4.89 ± 0.05^bA^
The potency of K_2_SiO_3_·nH_2_O (g L^−1^)	Ca^2+^ Concentration ((mg g^−1^ dwt) Salt levels (mM of NaCl)					
	Control (0 mM)		100		200	
	Leaves and stems	Roots	Leaves and stems	Roots	Leaves and stems	Roots
0	15.09 ± 0.62^aA^	8.40 ± 0.31^aA^	13.01 ± 0.54^bA^	5.35 ± 0.01^bB^	11.79 ± 0.75^bA^	5.32 ± 0.02^bA^
0.5	15.06 ± 0.58^aA^	7.15 ± 0.10^aB^	13.01 ± 0.09^abA^	6.48 ± 0.16^aA^	10.43 ± 1.28^bB^	5.45 ± 0.15^bA^
1.0	13.96 ± 0.13^abA^	6.83 ± 0.94^aB^	14.66 ± 0.25^aA^	5.52 ± 0.15^aB^	13.61 ± 0.62^bA^	5.10 ± 0.25^aA^

The values represent the means of three replicates. The means followed by the same letters (lower-case letters within lines and upper-case letters within columns, respectively) did not differ significantly at 5% probability by Tukey’s test.

**Table 4:** Photosynthetic rate (A), transpiration rate (E) and stomatal conductance (Gs) of Honeysuckle (*Lonicera japonica L.*) exposed to nine treatments.

**The potency of K_2_SiO_3_·nH_2_O (g L^−1^)**	**A (umol CO_2_ m^−2^s^−1^) photosynthetic rate Salt levels (mM of NaCl)**				
	**Control (0 mM)**	**100**		**200**	
	**Mean ± S.E**	**Mean ± S.E**	**Percent of control**	**Mean ± S.E**	**Percent of control**
0	15.13 ± 0.047^aA^	9.27 ± 1.22^bA^	61.27	3.03 ± 0.07^cB^	20.03
0.5	10.28 ± 0.09^aB^	5.74 ± 0.21^bB^	55.84	4.03 ± 0.03^cB^	39.20
1.0	6.98 ± 0.32^cC^	8.06 ± 0.81^bA^	115.47	9.55 ± 0.27^aA^	136.82
The potency of K_2_SiO_3_·nH_2_O (g L^−1^)	E (mmol m^−2^ s^−1^) transpiration rate Salt levels (mM of NaCl)				
	Control (0 mM)	100		200	
	Mean ± S.E	Mean ± S.E	Percent of control	Mean ± S.E	Percent of control
0	2.82 ± 0.36^aA^	0.86 ± 0.03^bA^	30.50	0.31 ± 0.01^cAB^	10.99
0.5	1.50 ± 0.19^aB^	0.42 ± 0.20^bB^	28.00	0.23 ± 0.01^bB^	15.33
1.0	1.02 ± 0.01^aC^	0.67 ± 0.11^bAB^	65.69	0.45 ± 0.06^cA^	44.12
The potency of K_2_SiO_3_·nH_2_O (g L^−1^)	Gs (mol m^−2^ s^−1^) stomatal conductance Salt levels (mM of NaCl)				
	Control (0 mM)	100		200	
	Mean ± S.E	Mean ± S.E	Percent of control	Mean ± S.E	Percent of control
0	0.16 ± 0.44^aA^	0.06 ± 0.00^bA^	37.5	0.02 ± 0.00^cA^	12.5
0.5	0.11 ± 0.02^aAB^	0.03 ± 0.00^bB^	27.27	0.01 ± 0.00^cA^	9.10
1.0	0.06 ± 0.00^aB^	0.05 ± 0.01^aA^	83.33	0.03 ± 0.01^bA^	50.00

The values represent the means of three replicates. The means followed by the same letters (lower-case letters within lines and upper-case letters within columns, respectively) did not differ significantly at 5% probability by Tukey’s test.

**Table 5:** Effect of K_2_SiO_3_·nH_2_O addition on the Si distribution within Honeysuckle (*Lonicera japonica L.*) plant under NaCl-stressed conditions.

**The potency of K_2_SiO_3_·nH_2_O (g L^−1^)**	**Si Concentration (mg g^−1^ dwt) salt levels (mM of NaCl)**					
	**Control (0mM)**		**100**		**200**	
	**Leaves and stems**	**Roots**	**Leaves and stems**	**Roots**	**Leaves and stems**	**Roots**
0	0.45 ± 0.00^aC^	0.08 ± 0.00^aC^	0.37 ± 0.00^bC^	0.02 ± 0.01^bC^	0.16 ± 0.00^cB^	0.00 ± 0.00^cC^
0.5	12.14 ± 1.08^aB^	3.75 ± 0.52^aB^	9.83 ± 0.76^bB^	2.09 ± 0.37^bB^	8.55 ± 1.02^bA^	1.29 ± 0.13^cB^
1.0	20.33 ± 2.96^aA^	5.40 ± 0.67^aA^	16.55 ± 2.41^aA^	5.00 ± 0.38^aA^	10.38 ± 1.23^bA^	3.00 ± 0.16^bA^

The values represent the means of three replicates. The means followed by the same letters (lower-case letters within lines and upper-case letters within columns, respectively) did not differ significantly at 5% probability by Tukey’s test.

**Table 6:** Effect of K_2_SiO_3_·nH_2_O addition on superoxide dismutase (SOD), peroxide (POD), and catalase (CAT) contents of Honeysuckle (*Lonicera japonica L.*) plant under NaCl-stressed conditions.

**The potency of K_2_SiO_3_·nH_2_O (g L^−1^)**	**SOD (Uμg-1 protein) Salt levels (mM of NaCl)**					
	**Control (0mM)**	**100**			**200**	
	**Mean ± S.E**	**Mean ± S.E**	**Percent of control**		**Mean ± S.E**	**Percent of control**
0	43.05 ± 7.13^bA^	49.91 ± 2.65^bC^	115.9		61.91 ± 1.26^aA^	143.8
0.5	43.77 ± 2.37^bA^	59.56 ± 1.87^aA^	136.1		62.82 ± 4.72^aA^	143.5
1.0	46.25 ± 1.31^cA^	54.32 ± 0.73^bB^	117.4		60.78 ± 5.76^aA^	131.4
The potency of K_2_SiO_3_·nH_2_O (g L^−1^)	POD (μg g^−1^ FW min^−1^) Salt levels (mM of NaCl)					
	Control (0 mM)	100		200		
	Mean ± S.E	Mean ± S.E	Percent of control	Mean ± S.E		Percent of control
0	82.22 ± 1.93^cB^	97.04 ± 0.14^bA^	118.0		137.0 ± 8.98^aA^	166.6
0.5	63.89 ± 0.28^cC^	82.96 ± 6.42^bB^	129.8		93.23 ± 1.68^aB^	145.9
1.0	86.67 ± 6.42^bA^	97.78 ± 3.58^aA^	112.8		100.0 ± 1.50^aB^	115.4
The potency of K_2_SiO_3_·nH_2_O (g L^−1^)	CAT (U mg^−1^ min^−1^) Salt levels (mM of NaCl)					
	Control (0 mM)	100			200	
	Mean ± S.E	Mean ± S.E	Percent of control		Mean ± S.E	Percent of control
0	0.37 ± 0.03^cA^	0.75 ± 0.03^aC^	202.7		0.61 ± 0.03^bB^	164.9
0.5	0.37 ± 0.03^bA^	0.96 ± 0.06^aA^	259.5		0.80 ± 0.11^aA^	216.2
1.0	0.37 ± 0.03^bA^	0.83 ± 0.02^aB^	224.3		0.77 ± 0.02^aA^	208.1

The values represent the means of three replicates. The means followed by the same letters (lower-case letters within lines and upper-case letters within columns, respectively) did not differ significantly at 5% probability by Tukey’s test.

## References

[b1] Shahbaz M., Ashraf M., Al-Qurainy F. & Harris P. J. C. Salt tolerance in selected vegetable crops. Crit Rev Plant Sci 31, 303–320 (2012).

[b2] Bybordi A. The influence of salt stress on seed germination, growth and yield of canola cultivars. Not Bot Horti Agrobo 38, 128–133 (2010).

[b3] Wang Z., Clifford M. N. & Sharp P. Analysis of chlorogenic acids in beverages prepared from Chinese health foods and investigation, *in vitro*, of effects on glucose absorption in cultured Caco-2 cells. Food Chem 108, 369–373 (2008).

[b4] Zhang B., Yang R., Zhao Y. & Liu C. Z. Separation of chlorogenic acid from honeysuckle crude extracts by macroporous resins. J Chromatogr B 867, 253–258 (2008). 10.1016/j.jchromb.2008.04.016 18456581

[b5] Shang X., Pan H., Li M., Miao X. & Ding H. *Lonicera japonic* Thunb.: Ethnopharmacology, phytochemistry and pharmacology of an important traditional Chinese medicine. J Ethnopharmacol 138, 1–21 (2011). 21864666 10.1016/j.jep.2011.08.016 PMC7127058

[b6] Leung H. W. C. *et al.* P38-associated pathway involvement in apoptosis induced by photodynamic therapy with *Lonicera japonica* in human lung squamous carcinoma CH27 cells. Food Chem Toxicol 46, 3389–3400 (2008). 18796326 10.1016/j.fct.2008.08.022

[b7] Yoo H. J., Kang H. J., Song Y. S., Park E. H. & Lim C. J. Anti‐angiogenic, antinociceptive and anti‐inflammatory activities of Lonicera japonica extract. J Pharm Pharmacol 60, 779–786 (2008). 18498715 10.1211/jpp.60.6.0014

[b8] Chen H. & Jiang J. G. Osmotic responses of Dunaliella to the changes of salinity. J Cell Physiol 219, 251–258 (2009). 19202552 10.1002/jcp.21715

[b9] Sushmita S., Priyanka D., Mamata R. & Surendra Chandra S. Osmolyte modulated enhanced rice leaf catalase activity under salt-stress. Adv Biosci Biotech 1, 39–46 (2010).

[b10] Demidchik V. & Tester M. Sodium fluxes through nonselective cation channels in the plasma membrane of protoplasts from Arabidopsis roots. Plant Physiol 128, 379–387 (2002). 11842142 10.1104/pp.010524 PMC148901

[b11] Demidchik V. & Maathuis F. J. Physiological roles of nonselective cation channels in plants: from salt stress to signalling and development. New Phytol 175, 387–404 (2007). 17635215 10.1111/j.1469-8137.2007.02128.x

[b12] Ebrahimi R. & Bhatla S. C. Ion distribution measured by electron probe X-ray microanalysis in apoplastic and symplastic pathways in root cells in sunflower plants grown in saline medium. J bioscience 37, 713–721 (2012). 10.1007/s12038-012-9246-y 22922196

[b13] Hilge M. Ca2+ regulation of ion transport in the Na+/Ca2+ exchanger. J Biol Chem 287, 31641–31649 (2012). 22822067 10.1074/jbc.R112.353573 PMC3442498

[b14] Jaleel C. A., Gopi R., Manivannan P. & Panneerselvam R. Antioxidative Potentials as a Protective Mechanism in Catharanthus roseus (L.) G. Don. Plants under Salinity Stress. Turk J Bot 31, 245–251 (2007).

[b15] Parida A. K., Das A. B. & Mittra B. Effects of NaCl stress on the structure, pigment complex composition, and photosynthetic activity of mangrove Bruguiera parviflora chloroplasts. Photosynthetica 41, 191–200 (2003).

[b16] Stepien P. & Johnson G. N. Contrasting responses of photosynthesis to salt stress in the glycophyte Arabidopsis and the halophyte Thellungiella: role of the plastid terminal oxidase as an alternative electron sink. Plant Physiol 149, 1154–1165 (2009). 19052149 10.1104/pp.108.132407 PMC2633845

[b17] Koyro H. W., Hussain T., Huchzermeyer B. & Khan M. A. Photosynthetic and growth responses of a perennial halophytic grass *Panicum turgidum* to increasing NaCl concentrations. Environ Exp Bot 91, 22–29 (2013).

[b18] Selmar D. Potential of salt and drought stress to increase pharmaceutical significant secondary compounds in plants. Landbauforsch Volk 58, 139–144 (2008).

[b19] Feng R. *et al.* Inhibition of activator protein-1, NF-κB, and MAPKs and induction of phase 2 detoxifying enzyme activity by chlorogenic acid. J Biol Chem 280, 27888–27895 (2005). 15944151 10.1074/jbc.M503347200

[b20] Lambert J. D., Hong J., Yang G. Y., Liao J. & Yang C. S. Inhibition of carcinogenesis by polyphenols: evidence from laboratory investigations. Am J Clin Nutr 81, 284S–291S (2005). 15640492 10.1093/ajcn/81.1.284S

[b21] Roche M., Dufour C., Mora N. & Dangles O. Antioxidant activity of olive phenols: mechanistic investigation and characterization of oxidation products by mass spectrometry. Org Biomol Chem 3, 423–430 (2005). 15678179 10.1039/b416101g

[b22] Zhang X. *et al.* Chlorogenic acid protects mice against lipopolysaccharide-induced acute lung injury. Injury 41, 746–752 (2010). 20227691 10.1016/j.injury.2010.02.029

[b23] Shan J. *et al.* Chlorogenic acid inhibits lipopolysaccharide-induced cyclooxygenase-2 expression in RAW264. 7 cells through suppressing NF-κB and JNK/AP-1 activation. Int Immunopharmacol 9, 1042–1048 (2009). 19393773 10.1016/j.intimp.2009.04.011

[b24] Ahmad R., Zaheer S. H. & Ismail S. Role of silicon in salt tolerance of wheat (*Triticum aestivum* L.). Plant Sci 85, 43–50 (1992).

[b25] Liang Y., Shen Q., Shen Z. & Ma T. Effects of silicon on salinity tolerance of two barley cultivars. J Plant Nutr 19, 173–183 (1996).

[b26] Liang Y. Effect of silicon on leaf ultrastructure, chlorophyll content and photosynthetic activity of barley under salt stress. Pedosphere 8, 289–296 (1997).

[b27] Parveen N. & Ashraf M. Role of silicon in mitigating the adverse effects of salt stress on growth and photosynthetic attributes of two maize (Zea mays L.) cultivars grown hydroponically. Pak J Bot 42, 1675–1684 (2010).

[b28] Al-aghabary K., Zhu Z. & Shi Q. Influence of silicon supply on chlorophyll content, chlorophyll fluorescence, and antioxidative enzyme activities in tomato plants under salt stress. J Plant Nutr 27, 2101–2115 (2005).

[b29] Epstein E. The anomaly of silicon in plant biology. P Natl Acad Sci 91, 11–17 (1994). 10.1073/pnas.91.1.11 PMC42876 11607449

[b30] Nayar P. K., Misra A. K. & Patnaik S. Rapid microdetermination of silicon in rice plant. Plant Soil 42, 491–494 (1975).

[b31] Giannopolitis C. N. & Ries S. K. Superoxide dismutases I. Occurrence in higher plants. Plant physiol 59, 309–314 (1977). 16659839 10.1104/pp.59.2.309 PMC542387

[b32] Chance B. & Maehly A. C. Assay of catalases and peroxidases. Methods Enzymol 2, 764–775 (1955). 10.1002/9780470110171.ch14 13193536

[b33] Gonzalo M. J., Lucena J. J. & Hernández-Apaolaza L. Effect of silicon addition on soybean (*Glycine max*) and cucumber (*Cucumis sativus*) plants grown under iron deficiency. Plant Physiol Bioch 70, 455–461 (2013). 10.1016/j.plaphy.2013.06.007 23845824

[b34] Yin L., Wang S., Li J., Tanaka K. & Oka M. Application of silicon improves salt tolerance through ameliorating osmotic and ionic stresses in the seedling of Sorghum bicolor. Acta Physiol Plant 35, 3099–3107 (2013).

[b35] Fadzilla N. M. & Burdon R. H. Salinity, oxidative stress and antioxidant responses in shoot cultures of rice. J Exp Bot 48, 325–331 (1997)

[b36] Mittal S., Kumari N. & Sharma V. Differential response of salt stress on *Brassica juncea*: Photosynthetic performance, pigment, proline, D1 and antioxidant enzymes. Plant Physiol Bioch 54, 17–26 (2012). 10.1016/j.plaphy.2012.02.003 22369937

[b37] Zollinger S. A. Performance constraints and vocal complexity in birdsong: Evidence from a vocal mimic. Ph.D thesis, Indiana University (2007).

[b38] Paranychianakis N. V. & Chartzoulakis K. S. Irrigation of Mediterranean crops with saline water: from physiology to management practices. Agr Ecosyst Environ 106, 171–187 (2005).

[b39] Gong H. J., Randall D. P. & Flowers T. J. Silicon deposition in the root reduces sodium uptake in rice (Oryza sativa L.) seedlings by reducing bypass flow. Plant Cell Environ 29, 1970–1979 (2006). 16930322 10.1111/j.1365-3040.2006.01572.x

[b40] Lemos Alves F. A., Ferreira Da Silva S. L., Da Silva E. N. & Gomes Da Silveira J. A. Clones of dwarf-precocious cashew submitted to salt stress and the accumulation of potassium and sodium. Rev Cienc Agron 39, 422–428 (2008).

[b41] Zhu J. K. Regulation of ion homeostasis under salt stress. Curr opin plant biol 6, 441–445 (2003). 12972044 10.1016/s1369-5266(03)00085-2

[b42] Szakiel A., Pączkowski C. & Henry M. Influence of environmental abiotic factors on the content of saponins in plants. Phytochem Rev 10, 471–491 (2011).

